# A dual therapy of off-pump temporary left ventricular extracorporeal device and amniotic stem cell for cardiogenic shock

**DOI:** 10.1186/s13019-017-0648-7

**Published:** 2017-09-07

**Authors:** Toshinobu Kazui, Phat L. Tran, Tia R. Pilikian, Katie M. Marsh, Raymond Runyan, John Konhilas, Richard Smith, Zain I. Khalpey

**Affiliations:** 1 Department of Surgery, Division of Cardiothoracic Surgery, University of Arizona, Tucson, AZ USA; 2 College of Medicine – Tucson, University of Arizona, Tucson, AZ USA; 3 Department of Physiological Sciences, University of Arizona, Tucson, AZ USA; 4 Department of Biomedical Engineering, University of Arizona, Tucson, AZ USA; 5 Artificial Heart Program, Banner University Medical Center, Tucson, AZ USA; 6 Division of Cardiohracic Surgery, Banner University Medical Center Tucson, 1501 N Campbell Ave., Rm 4302A, P.O. Box 245071, Tucson, AZ 85724-5071 USA

**Keywords:** Off-pump, Extracorporeal device, Left Ventricular Assist Device (LVAD), Refractory Cardiogenic Shock (RCS), Amniotic stem cell

## Abstract

**Background:**

Temporary mechanical circulatory support device without sternotomy has been highly advocated for severe cardiogenic shock patient but little is known when coupled with amniotic stem cell therapy.

**Case presentation:**

This case reports the first dual therapy of temporary left ventricular extracorporeal device CentriMag with distal banding technique and human amniotic stem cell injection for treating a severe refractory cardiogenic shock of an 68-year-old female patient. A minimally-invasive off-pump LVAD was established by draining from the left ventricle and returning to the right axillary artery with distal arterial banding to prevent right upper extremity hyperperfusion. Amniotic stem cells were injected intramyocardially at the left ventricular apex, lateral wall, inferior wall, and right subclavian vein.

**Conclusion:**

The concomitant use of the temporary minimally-invasive off-pump CentriMag placement and stem cell therapy not only provided an alternative to cardiopulmonary bypass and full-median sternotomy procedures but may have also synergistically enhanced myocardial reperfusion and regeneration.

## Background

Severe refractory cardiogenic shock (RCS) can be defined as significant left or right ventricular dysfunction resulting in sustained hypotension and hypoperfusion of end-organs. Treatment for RCS would include one or more modalities like pharmaco-agents (inotropes and vasopressors), intra-aortic balloon pump (IABP), revascularization in the case of myocardial infarction, and ultimately mechanical circulatory support (MCS) system to hemodynamically unload the heart and restore circulation. One of such MCS system is the centrifugal pumps like CentriMag (Thoratec Corp, Pleasanton, CA), Rotaflow (MAQUET Cardiopulmonary AG, Hirrlingen, Germany), or Sorin Revolution (Sorin Group USA, Inc., Arvada, CO). These temporary ventricular assist devices (VAD) have been widely used as bridge-to-decision or bridge-to-recovery in patients with refractory cardiogenic shock [1, 2]. However, cardiac recovery leading to VAD explantation is still observed in a very small population [3].

Most of patients who require this therapy fall into INTERMACS (Interagency Registry for Mechanically Assisted Circulatory Support) profile1. Conventionally, the temporary left VAD implantation has been performed through median sternotomy with or without cardiopulmonary bypass (CPB) support. These critically ill patients tend to bleed more during and after surgery for pre-operative liver dysfunction [4]. Especially for patient with previous sternotomy, the dissection can take longer and bleed more.

Patients with VAD may also be benefited from stem cell therapy (SCT), which has been shown to potentially increase circulatory perfusion [5, 6], promote angiogenesis and reduction of fibrin formation after VAD implantation [7], and improve ejection fraction (EF) [8]. More particularly, Nasseri et al. [5] looked at 10 patients on long-term left ventricular assist device (LVAD) concomitantly injected with bone marrow mononuclear cells. They found one improved cardiac function resulting in LVAD explantation, 2 deaths, and 7 others exhibited increased perfusion. However, these case reports and series are utilizing long-term LVAD support platform with maximal inflammation induction from median sternotomy; therefore creating an unfavorable environment for stem cell engraftment and differentiation that may potentially hinder cardiac recovery.

To recover the heart, a minimally invasive approach with stem cell therapy was applied to reduce surgical insult, avoiding CPB pump and median sternotomy. We cannulated at the apex of the left ventricle and returned through the right axillary artery with distal banding technique to avoid hyperperfusion. We then administered a bimodal delivery of amniotic stem cells with the intention of initiating cellular myocardial recovery.

## Case presentation

A 68-year-old female patient with past medical history of, two previous coronary artery bypass grafts, hypertension, chronic kidney disease, and diabetes mellitus, presented to a local hospital with chest pain and shortness of breath following an ST-elevation myocardial infarction. The patient was placed on an IABP and underwent a cardiac catheterization with the placement of five stents in the left anterior descending artery and saphenous vein graft. However she developed postoperative cardiogenic shock and multi-organ hypoperfusion despite her IABP and increased pressor requirements. She was then transferred to our institution for possible ventricular assist device placement.

Preoperative transesophageal echocardiography (TEE) demonstrated EF of 10% with apical akinesis, mild aortic insufficiency, moderate mitral and tricuspid valve regurgitation. Right heart was functional prompting for a CentriMag LVAD placement as Bridge-to-Decision/Recovery.

**Fig. 1:**
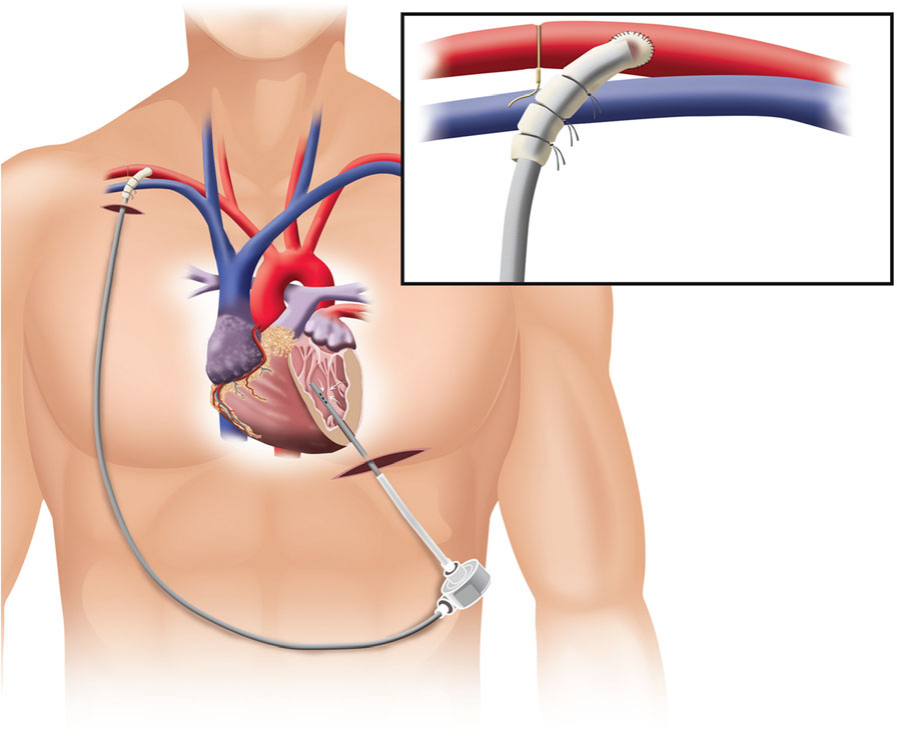
Application of the Thoratec® CentriMag® LVAD with inflow cannulation from the left ventricular apex, outflow cannulation via 8 mm Dacron graft into the right axillary artery, and arterial banding distal to outflow

The patient was placed supine position. The position of Left ventricular (LV) apex was confirmed by transthoracic echocardiography. A 5 cm mini-thoracotomy at 5th intercostal space was made. A pericardial well was created and A Thoratec 34 Fr inflow cannula (Thoratec Corp, Pleasanton, CA) was brought to the field. The Thoratec cuff was sewn into the left ventricular apex using plegetted 3–0 Ethibond sutures followed by a running 3–0 Prolene suture. The right axillary artery was exposed. Activated clotting time guided heparin was confirmed to be more than 200 s. An 8 mm Dacron graft was sewn into the axillary artery in end-to-side fashion using a 4–0 Prolene running suture. A 20 Fr EOPA cannula was passed through the graft with the tip extending to the level of the anastomosis. Silk ties were used to secure the cannula to the graft. The distal axillary artery was banded with a vessel loop. Banding prevented arterial and right axillary artery distal from over perfusion and ensured adequate cerebral perfusion. Remaining off-pump, the 34 Fr Thoratec inflow cannula was passed into the left ventricle. The position was confirmed by TEE. The cannula was secured to the cuff and was de-aired and connected to the CentriMag device (Fig. 1). The CentriMag was started and flow was established at 4.5 L/min. Human allograft and liquid matrix was inserted into the subclavian vein (2 times of 1cm^3^ of PalinGen Kardia Flow, Amnio Technology, Phoenix, AZ, USA) and into the anterior, lateral, and inferior walls of the LV (1cm^3^ of PalinGen Kardia Flow each).

Her end organ function recovered during the support. Transthoracic Echocardiography (TTE) was conducted which showed LV recovery, EF 30% with 1.5 LPM of LVAD flow at post op day 13. At post op day 20, fibrin clots developed in both the inflow and outflow cannula connectors prompting for an urgent decannulation at post op day 21. Upon off-pump LVAD removal, a subsequent IABP was placed and inotropic support was started in an effort to increase coronary perfusion. A head CT scan showed evidence of a small right sided ischemic cerebral infarct possibly due to clots dislodgement from the support/plumbing system or thrombotic occlusion of the right brachiocephalic trunk, right common carotid, right internal, and right vertebral arteries resulting in left sided hemiplegia. Extubation, IABP removal, and BiPAP administration was performed on post-explant day three. The patient was discharge to rehabilitation facility.

## Conclusion

With regard to implantable LVAD, off pump placement has been reported [9]. However, to our best knowledge, this is the first dual therapy of using a temporary off-pump implantation of a CentriMag as a left ventricular extracorporeal device with banding technique and human amniotic stem cell. CentriMag LVAD placement was performed in an off-pump fashion with left mini-thoracotomy and adjusted distal arm flow by placing a tourniquet. This is a viable short-term solution as bridge to decision/recovery especially for patients with previous sternotomy. Also, anastomosis of a graft to the right axillary artery can further prevent complications relating to direct axillary artery cannulation. This approach gave us a flexibility to extubate and to mobilize the patient while she was on the support. Avoiding the usage of CPB and full sternotomy can also minimize postoperative inflammation, preserve end organ function, and potentially prevent RV dysfunction [10].

One of the potential disadvantages of this approach was hyperperfusion of the right arm and right carotid artery. We were able to avoid the latter complication by tightening the axillary artery distal to the anastomosed site via a tourniquet such that the right radial pressure matched the left radial pressure upon stable support.

From the hemodynamic point of view, mechanically unloading the heart has been shown to increase survivability and promote differentiation of cardiac stem cells injected in the myocardium of an infarcted animal heart [11]; inhibit apoptotic mechanism and facilitated stem cells engraftment in a mouse model [12]. It appears that our minimally invasive off pump approach minimizes sternotomy-induced myocardial inflammation, reduces myocardial load, improves coronary perfusion such that a favorable environment was created for amniotic stem cell to engraft and resurrect the ailing heart. In fact, our patient’s left ventricle EF improved from 10 to 30% by postop day 13. This observed clinical improvement is concurrent to bone marrow stem cell studies reported by Gojo et al. [8] (EF improved from 6.4 to 40%) and Miyagawa et al. [13] (EF improved from 22 to 32%). Aside from safe to use, we think that direct stem cell injection may provide myocardial functional recovery but further study is highly warranted in the future.

The concomitant use of SCT and LVAD have been previously reported by Dib et al. [14] and Pagani et al. [15]. However, they were using skeletal myoblasts, which is less versatile than amniotic stem cells and long term LVAD, which can inflict maximal surgical insults. Our minimally-invasive off-pump CentriMag LVAD placement has the potential to achieve shorter recovery times and improve clinical outcomes in profound RCS and previous cardiac surgery patients. Finally, we further demonstrated that dual therapy of LVAD and SCT is also feasible, safe, and may be efficacious to LVAD explantation; thus moving toward a more prescriptive/individualized medical therapy for early and end stage heart failure patients.

## Abbreviations

CPB: CardioPulmonary Bypass
EF: Ejection fraction
IABP: Intra-aortic balloon pump
LV: Left ventricle
LVAD: Left ventricular assist device
MCS: Mechanical circulatory support
RCS: Refractory cardiogenic shock
SCT: Stem cell therapy
TEE: TransEsophageal Echocardiography
TTE: TransThoracic Echocardiography
VAD: Ventricular assist devices
